# Reorganizing the Multidisciplinary Team Meetings in a Tertiary Centre for Gastro-Intestinal Oncology Adds Value to the Internal and Regional Care Pathways. A Mixed Method Evaluation

**DOI:** 10.5334/ijic.5526

**Published:** 2021-02-25

**Authors:** Lidia S. van Huizen, Pieter U. Dijkstra, Patrick H.J. Hemmer, Boudewijn van Etten, Carlijn I. Buis, Linde Olsder, Frederike G.I. van Vilsteren, Kees (C.)T. B. Ahaus, Jan L.N. Roodenburg

**Affiliations:** 1 University of Groningen, University Medical Center Groningen, Department of Oral and Maxillofacial Surgery, Groningen, The Netherlands; 2 University of Groningen, University Medical Center Groningen, Department of Quality and Patient Safety, Groningen, The Netherlands; 3 Kerteza, a Worldwide Consultancy and Training Institute for Healthcare Organizations, Kasterlee, Belgium; 4 University of Groningen, University Medical Center Groningen, Center for Rehabilitation, Groningen, The Netherlands; 5 University of Groningen, University Medical Center Groningen, Department of Surgery, Groningen, The Netherlands; 6 University of Groningen, University Medical Center Groningen, Department of Gastroenterology and Hepatology, Groningen, The Netherlands; 7 Erasmus University Rotterdam, Erasmus School of Health Policy & Management, Rotterdam, The Netherlands

**Keywords:** oncology, integrated care, critical pathways (MeSH), care pathways, multidisciplinary team meetings (MDTM), added value, mixed method evaluation

## Abstract

**Introduction:**

The reorganisation of the structure of a Gastro-Intestinal Oncology Multidisciplinary Team Meeting (GIO-MDTM) in a tertiary centre with three care pathways is evaluated on added value.

**Methods:**

In a mixed method investigation, process indicators such as throughput times were analysed and stakeholders were interviewed regarding benefits and drawbacks of the reorganisation and current MDTM functioning.

**Results:**

For the hepatobiliary care pathway, the time to treatment plan increased, but the time to start treatment reduced significantly. The percentage of patients treated within the Dutch standard of 63 days increased for the three care pathways. From the interviews, three themes emerged: added value of MDTMs, focus on planning integrated care and awareness of possible improvements.

**Discussion:**

The importance of evaluating interventions in oncology care pathways is shown, including detecting unexpected drawbacks. The evaluation provides insight into complex dynamics of the care pathways and contributes with recommendations on functioning of an MDTM.

**Conclusions:**

Throughput times are only partly determined by oncology care pathway management, but have influence on the functioning of MDTMs. Process indicator information can help to reflect on integration of care in the region, resulting in an increase of patients treated within the Dutch standard.

## Introduction

Care pathways are accepted as a means to manage oncology care [1]. The management team of an oncological care pathway, tumour board, generally consists of a group of specialists that focus on 1) communication between different specialists on managing evidence-based treatment for oncology patients, 2) decision making in multidisciplinary team meetings (MDTMs) for oncology patients who need complex treatment plans and 3) multidisciplinary coordination of integrated care with timely start of treatment within the region [2,3,4]. MDTMs use digital medical records and clinical decision support systems in different ways [5,6]. MDTMs make a valuable contribution to the choice and planning of treatment [7,8,9] and lead to a better survival rate [10,11,12,13]. Consequently, MDTMs are considered the gold standard in oncology care pathway management [14,15,16,17,18,19,20,21] and the platform to accomplish clinical integration [22]. For optimal coordination and clear communication with patients, uniformity in working methods with standardised formats for MDTMs are advocated by European [23,24], Canadian [25] and American cancer treatment associations [26]. Additionally, MDTMs are also used for coordinating research, education, promoting and for diffusing best practices and new developments, so called ‘functional integration’ [22].

The Gastro-Intestinal Oncology (GIO) tumour board of our University Medical Centre (UMC) is a tertiary centre that organises oncology care together with partners in the northern region of the Netherlands and shares responsibility for optimising quality and improving the integration of care. This GIO tumour board manages care pathways for three groups of malignancies: colorectal, hepatobiliary and esophagus-stomach. In the Netherlands, the number of gastrointestinal cancer cases rose from 12,877 in 1989 to 23,985 in 2018, an increase of 86%. Especially the increase in fragile, elderly patients with gastrointestinal cancer led to a need for more complex care. This complexity led to lengthier discussions, longer MDTMs and longer throughput times for the patient to get a treatment plan. Given these trends, the UMC-GIO tumour board decided to reorganise the care pathways according to a previous developed model [27]. The aim of that reorganization was to make the care pathways more patient-centred, enabling shared decision making and to reduce throughput times to comply with the standards set by the Dutch Healthcare Inspectorate, formulated in the SONCOS standards (Stichting Oncologische Samenwerking: Council for Oncological Collaboration) [28]. The main interventions were: 1) immediate triage with direct ordering of missing diagnostics upon receival of the referral, 2) assessment of the patient before the MDTM in the outpatient clinic on the same day as the MDTM, 3) presence of the right specialisms during each MDTM to formulate an optimal multidisciplinary treatment plan and 4) seeing the patient shortly after the MDTM, on the same day, to share the proposal for treatment and decide together with the patient (shared decision making).

**Figure 1:**
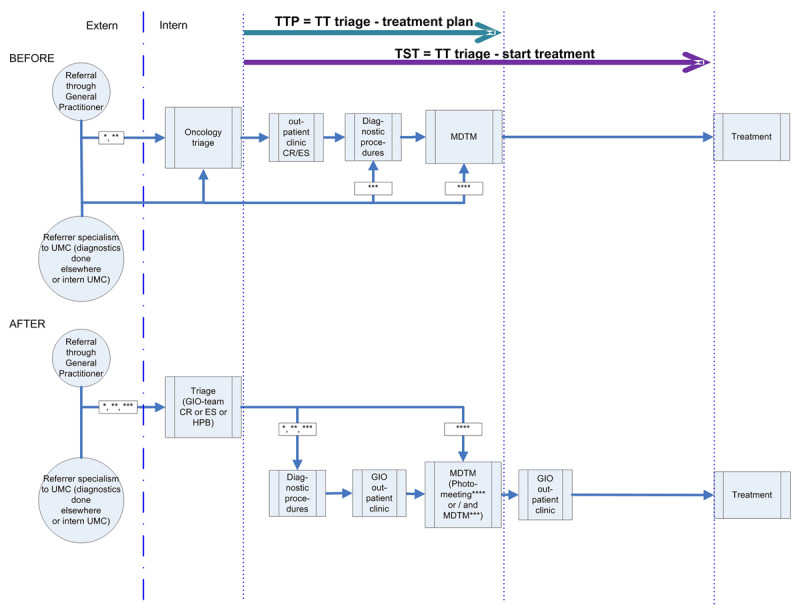
Before and after the reorganization with indicators. Legend: The green and purple arrows indicate TTP and TST respectively. For an explanation see the methods section Process indicators and study design. Abbreviations: GIO: Gastro-Intestinal Oncology, MDTM: Multidisciplinary team meeting; TTP: Time to Treatment Plan and TST: Time to Start Treatment, TT: Throughput Time, UMC: University Medical Centre. In the schematic arrows: *: Colorectal (CR), **: Esophagus-Stomach (ES), ***: Hepatobiliary (HPB) tertiary, ****: Hepatobiliary quaternary.

The care pathways start with referral to the UMC by a general practitioner or a specialist (tertiary or quaternary; Supplement 1). Before the reorganisation, patients following the colorectal and esophagus-stomach care pathways were seen at the oncology outpatient clinic before their treatment plan was discussed in an MDTM [29]. In several cases the diagnostic work-up was not yet complete. In the hepatobiliary care pathway usually images with a treatment plan were discussed at the MDTM before patients were invited to the oncology outpatient clinic. Due to the quaternary function, consultation ‘on paper’ is requested regularly and not all patients require to visit the UMC (e.g. a non-resectable tumour eligible for palliative chemotherapy can be handled by their local physician). As of April 2015, the triage with direct ordering of missing diagnostics was implemented. The first assessment of the patient in the outpatient clinic, GIO-intake, was on the same day as the MDTM in which their treatment plan was formulated (***Figure 1***). Decisions in the MDTMs are made by dedicated specialists involved in diagnostics and treatment for that GIO pathway. Directly after the MDTM, on the same day, the treatment options and consequences are explained to the patient. Specialisms involved in the treatment have the opportunity to speak with the patient. The reorganization did not change the role of the case managers, they plan the activities for diagnostic procedures and treatment in the same way.

When throughput times started to increase again, the GIO tumour board felt the need to evaluate the reorganisation by comparing its throughput times and the number of MDTMs per patient. In this study, we evaluated quantitatively the throughput times, number of hospital visits and number of MDTMs [11,30], and qualitatively the benefits and drawbacks of the reorganisation by interviewing specialists and case managers. This mixed methods approach sought to answer two questions:

1. What is the added value of the GIO-MDTM reorganisation in terms of throughput times, number of MDTMs and number of hospital visits?
2. What benefits and drawbacks do stakeholders of each care pathway perceive from the reorganisation of the GIO-MDTM and how could functioning of MDTMs be further improved?

## Methods

### Quantitative component

#### Sample size estimation

In a previous study on the effects of reorganising a care pathway for patients with head-and-neck cancers, data retrieved from 25 medical records before and 25 after a reorganisational intervention were sufficient to show a significant reduction in throughput times and hospital visits [31].

We therefore choose to analyse, for each care pathway, two sets of medical records, 25 before and 25 after the reorganisation. The first set included data on 25 consecutive patients referred at least four months before the start of the GIO-MDTM reorganisation, working back from December 31^st^ 2014. The other set included data who were referred four months after the reorganisation, i.e. from August 1^st^ 2015 onwards. Data were included on patients who were at least 18 years old and who had been discussed in a GIO-MDTM in our UMC. The following tumours were selected (ICD-O-03 ed1/ed3 [32]): esophagus C15, stomach C16, colon C18, rectum C209, pancreas C250, liver C220 and gall bladder C239. Data on patients treated for benign or neuroendocrine tumours were not included.

#### Process evaluation and study design

For process evaluation of the reorganization of GIO-MDTM, throughput times, the number of MDTMs per patient and the number of hospital visits were used as process indicators (i.e. quantitative outcome variables for this study). Throughput times were measured as the times from triage to the moment the treatment plan was available and to start treatment (***Figure 1***).

#### National standards

In assessing the added value, or efficiency, of the reorganisation we used modified SONCOS standards. The tertiary centre’s responsibility starts the moment the referral request is received and the centre obviously has no direct influence on the part of the care pathway before this referral. The standards state that, for patients with a GIO tumour, the throughput time for diagnostic procedures should be no more than 21 days; and that the throughput time from oncology intake, if referred to a tertiary treatment facility, to the start of primary treatment no more than 63 days. As the starting point for these throughput times, the standards take the day that the results of the biopsy, taken in the referring hospital, are known. Instead, we took timing of triage in our institution as starting day for throughput times. Thus, in this study, we set targets of 21 days for the time to get the treatment plan and 63 days for the time to start treatment (***Figure 1***).

Sometimes, tumour size was missing in the treatment plan. In these instances, we used Netherlands Cancer Registry data to retrieve missing tumour size data and to confirm dates we extracted from medical records.

#### Statistical analysis

To analyse whether the GIO-MDTM reorganisation had different effects for the different care pathways, a univariate general linear model analysis was performed. However, the assumptions for this type of analysis were not satisfied. Subsequently, several attempts were made to transform the data to meet the assumptions, but these failed because our data were too skewed. Instead we analysed effects of the reorganisation within each care pathway non-parametrically and report medians and interquartile ranges (IQR). Differences in age, gender, tumour localisation (ICD-O), tumour size, diagnostic type, treatment type and compliance with the 21-days standard and the 63-days standard, before and after the reorganisation of the GIO-MDTM, were analysed using Chi-Squared tests or Chi-Squared test exact if requirements were not met. Mann-Whitney-U tests were used to analyse throughput time differences. Statistical analyses were performed using SPSS 23.0 for Windows software. Statistical significance was set at 5%.

### Qualitative component

Semi-structured interviews were held with gate-keeping specialists and case managers from the three care pathways. The interviews focussed on perceived benefits and drawbacks, and the value of the reorganisation, the current functioning of the GIO-MDTM and how MDTMs could be further improved.

#### Interviews

During October and November 2019, three surgeons, three gastroenterologists and three case managers were interviewed. After receiving their verbal informed consent, semi-structured interviews started with providing information on the quantitative results of this study. The interview continued with the question: ‘What do you think is the role of the gate-keeping specialist / case manager in a GIO-MDTM?’. The interviewer used a topic list as interview guide (Supplement 2). Interviews lasted 25 to 40 minutes, were audio recorded and transcribed.

#### Thematic analysis

Quotes were extracted from the transcripts. The participants were asked to review and confirm their personal transcripts and extracted quotes. Quotes were then anonymised. In the first stage of the inductive analysis [31,32], codes were given to quotes related to the reorganisation of the GIO-MDTM and its current functioning [30,33,34,35]. The codes were placed in a coding tree in relation to the research question with three main themes: planning for integrated care, added value of the MDTM and the management of the care pathway (Supplement 3) [36,37]. Thereafter a second coder gave quotes codes from the coding tree. Codes were judged as either being a benefit or a drawback that could be improved. Disagreements in coding between the coders and the researcher were discussed. After the preliminary results were collated, a member check was performed to ensure credibility [38].

## Results

### Quantitative analysis

In total, data from 194 medical records were included in this study; 96 before and 98 after the reorganisation (Supplement 4: Tables a-c). All groups had at least 25 patients that started treatment. A data check revealed that 3% of the data were not in accordance with the Netherlands Cancer Registry and were changed accordingly. The throughput times based on the Netherlands Cancer Registry database were shorter than those based on medical records (mean difference 0.5 days). Staging verification showed no differences for the tumour sizes. Mean (sd) age of patients before and after the reorganisation was 66.2 (9.3) respectively 65.4 (12.5) years. In all the pathways, tumours were somewhat larger after the reorganisation. Outliers were explored and, in most cases, comorbidity induced extended throughput times.

In the colorectal care pathway, after the reorganisation, the number of hospital visits in the period from triage to start of treatment tended to increase (p = .092) (***Table 1*** and ***Figure 3a***). Nevertheless, the standards for throughput times from triage to get the treatment plan and from triage to start treatment were met for a higher proportion of patients after the reorganisation (85 vs 93%).

**Table 1:** Throughput times, number of MDTMs and hospital visits before and after the reorganisation in the different pathways.


	COLORECTAL			HEPATOBILIARY			ESOPHAGUS-STOMACH		

	BEFORE	AFTER		BEFORE	AFTER		BEFORE	AFTER	

Number of patients diagnosed*	2014 (n = 32)	2015 (n = 34)	p	2014 (n = 36)	2015 (n = 32)	p	2014 (n = 28)	2015 (n = 32)	p

	Median (IQR)	Median (IQR)		Median (IQR)	Median (IQR)		Median (IQR)	Median (IQR)	

Throughput time (days)									

TTP triage – treatment plan	8.0 (4.3;26.8)	13.0 (6.0;22.5)	.653	5.5 (1.0;12.0)	9.0 (5.0;22.0)	.035	15.0 (7.0;23.0)	13.0 (5.3;19.0)	.292

treatment plan within 21 days**	66%	74%	.485	89%	72%	.075	71%	87%	.370

TST triage – start treatment	37.5 (24.3;55.8)	34.0 (24.0;46.0)	.663	58.0 (46.5;68.5)	47.0 (37.0;54.0)	.026	31.0 (22.5;41.5)	28.0 (20.5;39.5)	.521

start treatment within 63 days**	85%	93%	.420	60%	88%	.024	96%	100%	1.000

Number of MDTMs	1.0 (1.0;2.0)	1.0 (1.0;2.0)	.307	1.0 (1.0;1.0)	1.0 (1.0;2.0)	.026	2.0 (1.0;2.0)	1.0 (1.0;2.0)	.079

Number of patients treated***	2014 (n = 26)	2015 (n = 27)		2014 (n = 25)	2015 (n = 25)		2014 (n = 25)	2015 (n = 25)	

Number of hospital visits									

triage – treatment plan	2.0 (1.0;3.0)	3.0 (2.0;4.0)	.092	1.0 (0.0;3.0)	1.0 (1.0;3.0)	.027	4.0 (2.0;5.8)	3.0 (2.0;4.0)	.037

triage – start treatment	3.5 (3.0;5.0)	5.0 (3.0;6.0)	.157	3.0 (2.0;4.0)	3.0 (2.0;4.0)	.933	5.5 (4.0;7.0)	5.0 (3.0;6.0)	.238


Legend: Abbreviations: IQR: Inter Quartile Range (25^th^ and 75^th^ percentiles), MDTM: Multidisciplinary team meeting, TST: time to start treatment, TTP: time to treatment plan; *: number of patients consecutively discussed in the MDTM in the UMC of the care pathways. Differences between before and after the reorganisation were tested using Mann-Whitney-U, except the percentages **: ‘treatment plan within 21 days’ and ‘treatment within 63 days’ were tested using with Chi^2^ test, ***: number of patients discussed in the UMC MDTM that were treated in the UMC or in the region.

**Figure 2:**
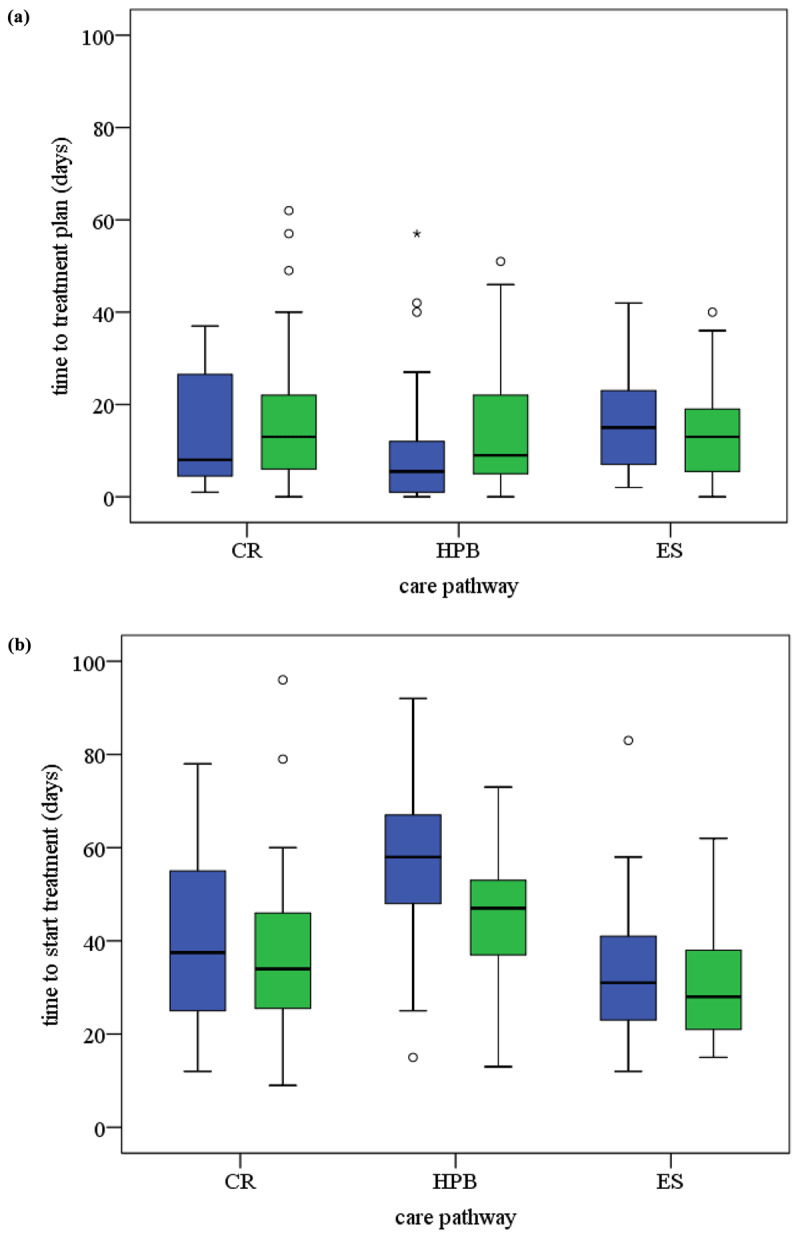
**(a)** Box and whisker plots time to treatment plan. **(b)** Box and whisker plots time to start treatment. Legend: CR: colorectal, HPB: hepatobiliary, ES: esophagus-stomach; TST: time to start treatment; TTP: time to treatment plan. Blue is before and green is after the MDTM reorganisation; ^O^: outlier, *: outlier Tukey’s method IQR; IQR: Inter Quartile Range.

In the hepatobiliary care pathway, more primary tumours were treated after the reorganisation (p = .039) (Supplement 4: Table b), the time to get the treatment plan increased (p =.035) but the time to start treatment decreased (p = .029) (***Table 1*** and ***Figure 2a***). The number of hospital visits between triage and treatment plan increased (p = .027), and more MDTMs were needed to come to a treatment plan (p = .026) after the reorganisation. After the reorganisation fewer patients got their treatment plan within 21 days. The percentage of patients that started their treatment within 63 days increased to 88% (p = .024).

**Figure 3:**
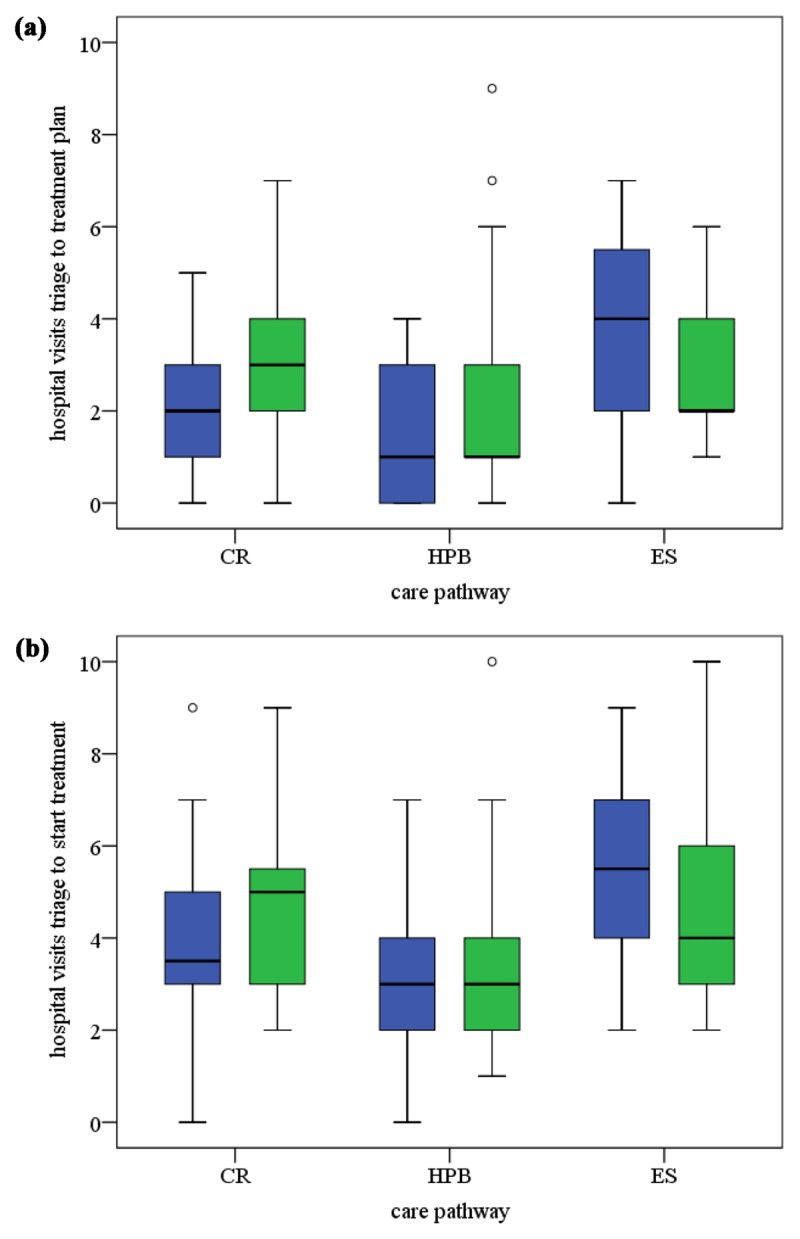
**(a)** Box and whisker plots number of hospital visits from triage to treatment plan. **(b)** Box and whisker plots number of hospital visits between triage and start treatment. Legend: hospitals visits per patient (CR: colorectal, HPB: hepatobiliary, ES: esophagus-stomach). Blue is before and green is after the MDTM reorganisation; ^O^: outlier.

In the esophagus-stomach care pathway, patients in our post-reorganisation sample were older than those in the pre-reorganisation sample (p = .050) and the number of hospital visits needed to come to a treatment plan was less after the reorganisation (p = .037). The number of MDTMs per patient tended to decrease (p = .079; ***Table 1***). The percentage of patients that started their treatment within 63 days increased and in 2015 the standard of 63 days was met for all.

### Qualitative analysis

From the transcripts, 251 quotes were extracted. In total 50 codes (Supplement 3) were identified related to the reorganisation of the GIO-MDTM and its current functioning. These codes were given 630 times (Supplement 3). Inter-coder agreement was 62.5%. Codes representing a benefit (30 codes identified, 418 times) were given twice as often than those representing a drawback (20 codes, 212 times). The 10 most frequently given codes were given to 56 % of the 251 quotes.

During a thematic synthesis, three main themes emerged from the data; 1) increase of the added value of the MDTMs, for example availability of expert specialisms had increased, 2) greater focus in the planning on continuity and integration of care, for example planning in cooperation with other regional hospitals had improved, 3) greater awareness that improvements could be made in the management of GIO care pathways, such as using a dashboard to monitor ‘real time’ relevant throughput times for GIO patients on the hospital’s MDTM registration list.

#### The added value of the GIO-MDTMs (codes 17–34)

Most interviewees regarded a GIO-MDTM as the moment where all expertise comes together to decide an optimal multidisciplinary treatment plan. A gastroenterologist explained:

“The value of the MDTM is twofold: 1) for the patient who visits the GIO outpatient clinic, you have thought carefully about the possible diagnosis and multidisciplinary treatment (code 24) 2) it is good for the cohesion within the team, to know your colleagues with whom you work well, which means that you can also find each other easily in other circumstances.” (code 18).

During a GIO-MDTM, the gate-keeping specialism for each patient is responsible for the quality of the intake and presents their patients. That specialism thus plays a key role for patients and also for colleagues. In addition, the chair of the GIO-MDTM also fills an important role. The chair has to monitor and guide the meeting process, summarise discussions and formulate the conclusions. The chair needs to distinguish non-complex cases, or ‘formalities’, from complex cases to ensure an efficient discussion. A surgeon said:

“As chair, I prepare for a meeting thoroughly. I review the patients to estimate the time needed for each one: a ‘formality’ or an extended discussion.” (code 26).

Each care pathway had different dynamics reflecting differences in the biology of the tumours. Although participants noted that it is important to prepare for the MDTM, most specialisms did not schedule time for this. A surgeon said:

“It is both time consuming and important for a chair to prepare well for the MDTM, but no time is scheduled for this the day before our MDTM.” (code 23).

The participants stated that good preparation makes the MDTM more efficient for all persons present and it is good for patient care. A case manager said:

“Everybody wants time to reflect on their own preparation for the MDTM, because it is their patient being presented who needs an optimal treatment plan.” (code 32).

#### Focus in planning on continuity and integrated care (codes 1–16)

The case managers played a distinct role in the care pathway. They focused on all patients’ needs, including psychosocial aspects. They aim to speed up the diagnostic process by getting information from the referrer where possible and, during that process, they stay in contact with the patient, the referring hospital and the treating specialist, signalling problems in throughput times and acting to prevent delays when possible. A case manager said:

“The role of the case manager is to prepare the agenda for the MDTM and to act upon decisions of the MDTM.” (code 8).

A surgeon member of a tumour board put it like this:

“We steer tightly, using the case manager to acquire diagnostic results from the periphery on time. A few times, the results had not arrived on time, but we decided to discuss the patient at the MDTM with the information at hand.” (code 11).

The latter part of this quote reflects a dilemma we heard several times: helping the patient is more important than a perfect process in the hospital. Another aspect of the case manager’s focus on the patient and on integrated care was that they implemented an improvement shortly after the reorganisation of the GIO-MDTM. Patients had commented that they understood the diagnosis and the treatment plan, but that the explanation of the different treatment options and consequences was too much for them to digest in a single hospital visit.

#### GIO care pathways management and improvement awareness (codes 35–50)

Most interviewees stated that further improvements could be made, but that finding time to reflect and gain support to implement improvements was difficult. Throughput times cannot always be influenced by a physician or care pathway management. The available time in the operating theatre is in part determined by the capacity of the anaesthesiology department. A gastroenterologist said:

“The throughput time of 6–8 weeks for an Endoscopic Retrograde Cholangiopancreatography is determined by the sedation capacity of [the department of] anaesthesiology.”. A dashboard with indicators was seen as potentially helpful. A surgeon member of a tumour board said:

“We should have a dashboard to monitor our registration list for the GIO-MDTM in relation to relevant throughput times.” (code 46).

Another aspect highlighted was that not all parties involved in the GIO-MDTM were invited to meetings where policy and improvement opportunities were discussed. A case manager said:

“A tumour board manages our care pathway. As a case manager or nursing consultant, you are not invited to the policy meetings.” (code 45).

## Discussion

### Quantitative results

After the reorganisation, throughput times to start treatment decreased significantly but throughput times to get the treatment plan increased in the hepatobiliary pathway. In the two other pathways, the percentages of cases meeting the 21-day standard set for the treatment plan increased somewhat but not significantly. In all the pathways, a higher percentage of cases met the standard to start treatment within 63 days, but only significantly in the hepatobiliary pathway. The number of MDTMs increased significantly in the hepatobiliary pathway. The number of hospital visits from triage to treatment plan increased significantly in the hepatobiliary pathway but decreased significantly in the oesophagus-stomach pathway.

The reorganisation aimed to reduce throughput times by standardising the work for the majority of non-complex patients and thereby gaining time to discuss the more complex cases. In the UMC, as a tertiary and quaternary centre, an increasing number of older patients with more comorbidities are seen, which explains an increase in larger tumours. Generally complex patients with advanced diseases benefit most from MDTM discussions, also described as the ‘Flying Dutchman phenomenon’ blown from one site-specific MDTM to another until finally reaching safe haven [29]: patients getting the best possible treatment plan through a multidisciplinary approach in a tertiary centre [12,29,39,40]. Developments required more intensive discussion and coordination between professionals and this is reflected in increased throughput times and number of hospital visits from triage to treatment plan in the hepatobiliary pathway. During the reorganization there were no task shifts from doctors to nurses or to general practitioners. An explanation for the decrease in throughput time from triage to start treatment in the hepatobiliary pathway (a 9-day difference in median times), despite a longer throughput time from triage to treatment plan, could be improved case coordination as a result of the reorganisation of the MDTM. Given the increasing percentage of complex cases, we argue that the SONCOS standards are too strict in expecting throughput times to be met for all patients. Indeed, for head-and-neck cancer patients in the Netherlands [41,42], there has been a modification, now expecting 80% of patients to meet the time to start treatment. Therefore, we would recommend healthcare policymakers to set throughput time standards but expect hospitals to only meet these for about 75% [43,44].

In the hepatobiliary pathway, before the reorganisation, patients were not seen in the outpatient clinic before the MDTM and decisions were taken based on imaging and documents. After the reorganisation, patients were seen before the MDTM, and additional hospital visits were scheduled to prepare for the treatment. This change resulted in longer throughput times and an increase in the number of MDTMs. Recently a re-evaluation project was started with the region to optimize the care pathway including the development of a dashboard.

In the colorectal care pathway, the number of hospital visits also tended to increase after the reorganisation. Intake and assessment by different specialties on the same day as the GIO-intake resulted in an overwhelming amount of information being presented to the patient. It was therefore decided to arrange an additional visit to explain the medical situation and the alternative treatments to the patient and their supporters. For such patients, efficiency has its limits: they need time for explanation and reflection in order to make a ‘well-weighted, shared decision’ with their treating specialist e.g. in an elderly MDTM [45].

Conversely, for the esophagus-stomach care pathway, the number of MDTMs tended to decrease as well as the number of hospital visits needed to come to a treatment plan. Another improvement was seen in the integration of surgical capacity. Here, since January 2019, a secondary hospital in the region shares its surgical capacity with the UMC’s GIO centre for stomach surgery. The MDTMs held by UMC and by the secondary hospital have been merged and using video-conferencing to reduce the number of MDTMs and decrease throughput times. Research on care pathway management in Scotland has shown that throughput time measurements on several levels should be taken into account to improve coordination in a region [46], and this is reflected in our recommendations below.

### Qualitative results

Twice as many codes were annotated as benefits than as drawbacks for the functioning of the GIO-MDTM. However, some of the benefits were already experienced as an advantage of having MDTMs before the reorganisation. From the interviews, it became clear that, following the reorganisation, the value of the MDTMs had increased. The different treatment modalities were better discussed between the appropriate specialisms with more attention to patient wishes. This was largely caused by availability of all expertise at the meeting to discuss complex cases and to cooperate in a multidisciplinary way in formulating an optimal treatment plan for individual patients. In this way, the reorganisation enhanced quality and integration of care for the three patient groups and, what is more, the interviewees said that the reorganised MDTMs also improved interpersonal relations between participants. These improvement contributed positively to discussions and resulted in better treatment plans. These findings are in line with previous study findings [47,48]. Another observation was the improvement in case coordination due to the more complete presence of required disciplines during the MDTM and the better relationships. Although the importance of improved case coordination between healthcare professionals with better interpersonal relationships has also been found previously [49,50,51,52], more research is needed to understand the underlying processes and the way it adds value to a care pathway.

Case managers believed that throughput times to get the treatment plan and throughput times to start treatment could be further reduced through stricter monitoring of the completeness of the diagnostic information needed to start treatment. The importance of strict monitoring has been identified elsewhere [53,54] but we noticed that the ‘circle of influence’ of a care coordinator or case manager is limited. The case manager has no control over or mandate for discipline-bounded capacities such as slots for diagnostic procedures. Such a mandate depends on the leadership and style of communication in the tumour board and the MDTM.

From the interviews, it became clear that the GIO-MDTMs would benefit from participants being better prepared. Specialists within the same department could discuss treatment possibilities from their perspective before the MDTM, and prepare questions to discuss with other specialists to optimise the proposed treatment. In general, there is no preparation time scheduled for the MDTM participants. The chair should be well prepared, and should earmark time for the different disciplines, so that discussions within a discipline during an MDTM would then take less time and the MDTM would be more efficient. Surgical oncologists elsewhere have reported that MDTM members have good insight into their own multidisciplinary team performance and state that all MDTMs would benefit from good leadership, good preparation of MDTMs and appropriate presentation of information by the gate-keeping specialists [55,56,57,58].

All participants of the GIO-MDTMs were highly motivated to improve efficiency of the meetings but they experienced a lack of time to prepare the meetings. Although the UMC, as a tertiary centre, treats mainly the more complex cases, there are sufficient surgical treatments to meet the SONCOS indicator for the ‘number of surgical cases’, which is an indicator for being a ‘competent’ surgeon [28]. However, this indicator should not be seen as justification for adversely affecting the time available for participants to prepare for an MDTM. Additionally, there remains a dilemma for the hepatobiliary pathway. The efficiency of the care pathway in terms of diagnostic procedures against the importance of meeting the patient before making a treatment plan at the MDTM so that the patient’s wishes concerning treatment can get more attention and can be optimally included [59].

### Combining quantitative and qualitative results

The interviews provided an insight into the complex dynamics of oncology care pathways and the functioning of their MDTMs. Collaboration in an MDTM is not only about efficiency and indicators like throughput times, but also about cooperation, respect for other team members and the commitment of all team members, and good leadership [12,48,60].

The importance of evaluating interventions in oncology care pathways is shown, including detecting unexpected drawbacks. This study showed the importance of evaluating adjustments or interventions in internal and regional care pathways in order to detect any unexpected drawbacks, to structure continuous improvement [43,61] and to organize care pathways in an integrated way. This mixed method approach, provides insight into how an oncology care pathway operates, the contribution of the individual members, their appreciation and assessment of the cooperation [62].

### Limitations of this study

A limitation of this study is the lack of generally accepted indicators for care pathway management and definitions of those indicators that do exist [46,57,63]. We modified Dutch SONCOS standardised indicators to evaluate the reorganisation of the care pathways in order to be comparable to the indicators used in earlier research on the care pathway of head-and-neck cancer patients [31]. Contrary to our expectations, we did not find a significant decrease in throughput times for the different GIO care pathways. We saw that the clinical presentation, the biological behaviour of tumours, types of treatment and treatment combinations differed considerably from the care pathway of head-and-neck cancers. Further, we noted that the UMC’s focus increasingly on the care of complex patients with larger tumours, that the incidence of tumours in the elderly is increasing, and that these factors may be important confounders in not finding a significant change following reorganisation.

### Recommendations

Based on the results of our study, we formulated the following recommendations

1. Make a policy plan with the region, for a specific period with accurate, recent performance data and reflect on possibilities to improve the care pathway (code 17).
2. Create a team of people who know and trust each other, who promote interaction and commitment using a U-form table in their meeting rooms (code 44) where colleagues can confront each other respectfully about desirable and undesirable behaviours (code 18).
3. Ensure all specialist disciplines attend the MDTM (code 24 and code 25) to formulate the best treatment plan for each patient, including customisation for complex or comorbid cases (code 10).
4. Make medical and psychosocial information available during MDTMs (code 31) and include patient wishes in the treatment plan e.g. by planning an elderly MDTM before the treatment MDTM (code 14).
5. Provide clarity on everybody’s individual role, before, during (code 22) and after the meeting to optimise time management during the MDTM (code 30).
6. The chair should show leadership and motivate the team by taking responsibility for directing the discussion in the meetings and summarise the conclusions and formulate the treatment plans according to the format in the guidelines (code 26).
7. Provide all MDTM participants with dedicated time to prepare for the meeting (code 23) since this will increase meeting efficiency and the quality of the treatment plan (code 22).
8. Set up an integrated dashboard to monitor relevant real time indicators for your care pathway, such as ‘throughput time differences from standard’ or hospital visits, and evaluate the performance (code 46).

The results and recommendations show that improving performance requires an improved functioning of MDTMs (clinical integration), participation of all specialists with clear roles (professional integration), resources such as time, sufficient performance information and quality improvement efforts (functional integration), a regional policy (organizational integration) and shared commitment and mutual trust to improve the performance of the pathway (normative integration) [22].

However ‘real time’-dashboard implementation is complicated for functional integration in a care pathway, but is currently under development.

### Further Research

To justify the existence of time-consuming events such as MDTMs in oncological care pathways, it is important to measure their added value. Further research could be directed at investigating the value of real time dashboard information, and consider the waiting times and the status of diagnostic procedures in reaching a personalised treatment plan in an MDTM. On the tumour board level, further research could focus on what indicators enable effective care pathway management. For example, indicators that 1) present real time throughput time information on diagnostic procedures and treatment steps, 2) enable informed decision-making on diagnostic and therapeutic capacity and 3) increase efficiency by reducing non-value adding diagnostic procedures or treatments.

## Conclusions

Reorganising the GIO-MDTM and outpatient clinic had different effects on each care pathway. For the hepatobiliary pathway, the throughput time from triage to treatment plan increased, but the throughput time from triage to start treatment reduced. No other significant changes were identified. Overall, the percentage of patients treated within the Dutch standard of 63 days increased.

The efficacy of an integrated multidisciplinary care pathway needs constant attention. It can be assessed with a mixed method approach. Beside results of quantitative evaluation like throughput times, a qualitative approach is recommended for assessment of the human factor in cooperation between different disciplines.

## Data Accessibility Statement

Datasets will be available from the corresponding author on reasonable request.

## Additional Files

The additional files for this article can be found as follows:

10.5334/ijic.5526.s1 Supplementary File 1. Illustration GIO care pathway before the reorganization.

10.5334/ijic.5526.s2 Supplementary File 2. Interview Guide.

10.5334/ijic.5526.s3 Supplementary File 3. Coding tree reorganization GIO-MDTM.

10.5334/ijic.5526.s4 Supplementary File 4. Patient and tumour characteristics of the care pathways.

## Patient involvement in study design

Patients did not participate in the study design because the main purpose of the study was to evaluate the reorganization of the GIO-MDTM on efficiency in the care pathways for healthcare professionals.

## Author information

The University Medical Center Groningen (UMCG) is developing patient-centred, integrated care pathways for various patient groups. In addition to medical and logistic aspects, laws and regulations for quality and patient safety are included. The UMCG has an institutional ISO 9001 for Healthcare certificate and ISO 27001 Information Security certificate for their care, research and educational processes. The Quality and Patient Safety research group evaluates the implementation of care pathways and MDTMs to develop indicators for the management level of care pathways led by healthcare professionals. The four leading researchers for this paper are LvH, PUD, KA and JR. Of these, LvH works as a consultant for quality and patient safety for different care pathways seeking to implement improvements and is involved in certification of these care pathways at the regional level. PUD works as a researcher in the field of rehabilitation and is an epidemiologist. KA chaired the Quality and Patient Safety research group of the UMCG and now chairs the Health Services Management & Organisation Department of the Erasmus School of Health Policy & Management. His research interest is quality improvement and value-based healthcare. JR was chair of the multidisciplinary Head and Neck Oncology Group of the UMCG for 30 years and chair of the Dutch Multidisciplinary Head and Neck Oncology Group for 8 years. The centralisation of head and neck cancer care in eight centres was achieved under his leadership in 1993.
