# Supplementary material for: Reorganizing the Multidisciplinary Team Meetings in a Tertiary Centre for Gastro-Intestinal Oncology Adds Value to the Internal and Regional Care Pathways. A Mixed Method Evaluation
Source: Int J Integr Care. 2021 Feb 25;21(1):8. doi: 10.5334/ijic.5526 (PMC7908930; doi:10.5334/ijic.5526)
Supplement: Supplementary File 1. — Illustration GIO care pathway before the reorganization. [file ijic-21-1-5526-s1.pdf]

Supplementary file 1: Illustration GIO Care pathway before the reorganization

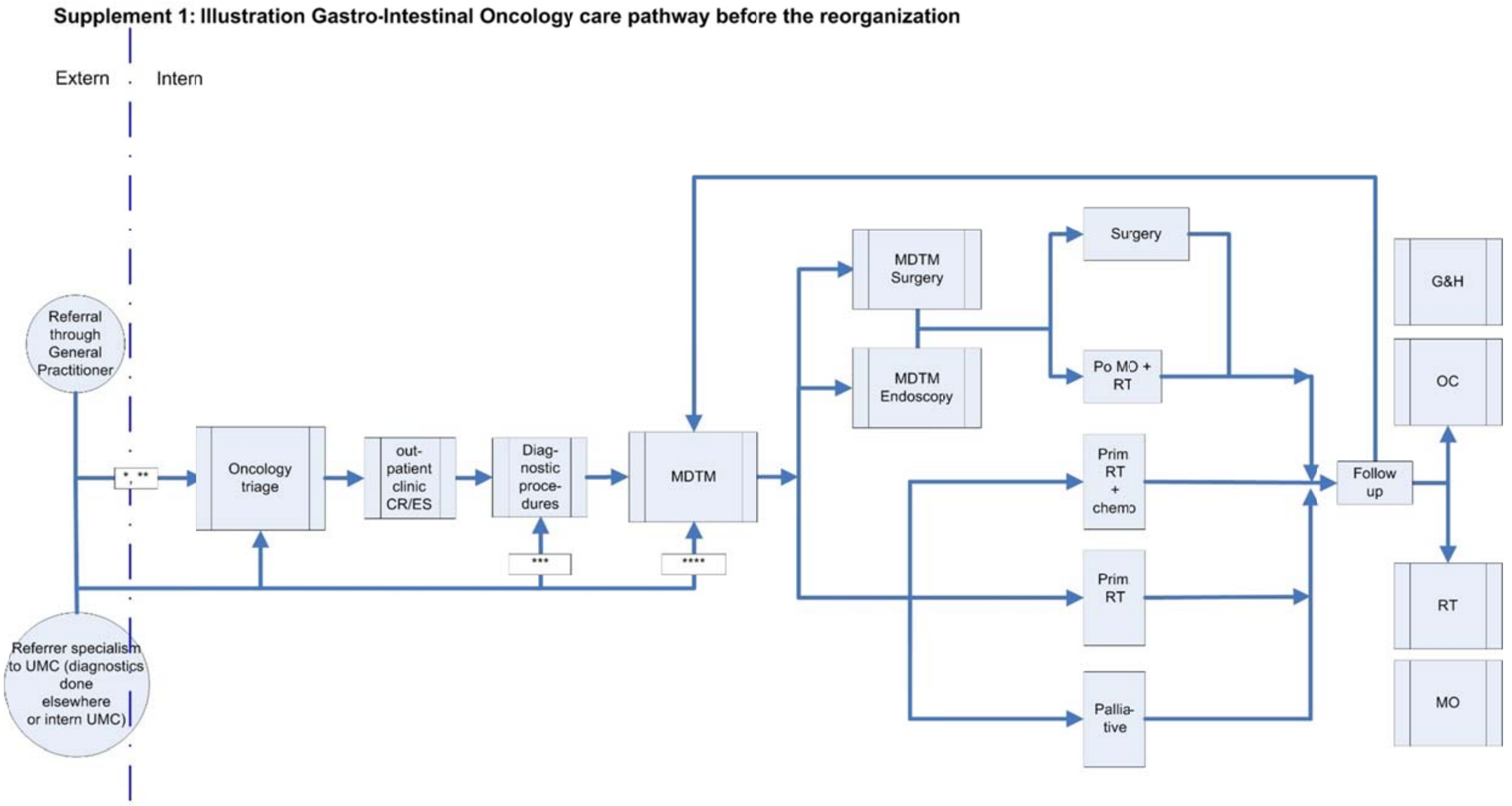

Legend Supplement 1: The care pathways start with referral to the UMC by a general practitioner in case of population screening for bowel disease or a specialist (tertiary or quaternary). During the oncology triage the diagnostic work is reviewed before a patient is invited to the outpatient clinic and discussed in the MDTM. After the GIO-MDTM additional MDTMs are needed to prepare for a specific treatment, e.g. MDTM for surgery and Endoscopic Retrograde Cholangiopancreatography. After the treatment the gate-keeping specialism performs a short follow-up before the patient is transferred back to the referring hospital or general practitioner.
Abbreviations: Chemo: Chemotherapy, G&H: Gastroenterology & Hepatology, GIO: Gastro-Intestinal Oncology, MDTM: Multidisciplinary team meeting, MO: Medical Oncology OC: Oncology surgery, Po: Preoperative, Prim.: Primary, RT: Radiotherapy, UMC: University Medical Centre.
In the schematic arrows: *: Colorectal, **: Esophagus-Stomach, ***: Hepatobiliary tertiary, ****: Hepatobiliary quaternary.
