# Supplementary material for: Reorganizing the Multidisciplinary Team Meetings in a Tertiary Centre for Gastro-Intestinal Oncology Adds Value to the Internal and Regional Care Pathways. A Mixed Method Evaluation
Source: Int J Integr Care. 2021 Feb 25;21(1):8. doi: 10.5334/ijic.5526 (PMC7908930; doi:10.5334/ijic.5526)
Supplement: Supplementary File 2. — Interview Guide. [file ijic-21-1-5526-s2.pdf]

Supplementary file 2: Interview Guide

Topic	Questions
Results interpretation	These are the results of the evaluation measures: throughput times, MDTMs and hospital visits. Can you describe the relevance of these results for you? What is your impression of the reorganisation of the MDTM in your care pathway?
Role of gate-keeping specialist or case manager	What is the role of the gate-keeping specialist / case manager in the GIO-MDTM?<br>Holding MDTMs is required by the SONCOS guidelines and the Dutch Health Care Inspectorate, how useful do you think MDTMs are with your patient category?
Added value	What would be an ideal GIO-MDTM?<br>What do you think could be modified in the GIO-MDTM to make the consultation more effective and more efficient?<br>How are MDTMs evaluated?

GIO: Gastro-Intestinal Oncology, MDTM: Multidisciplinary team meeting, SONCOS: Stichting Oncologische Samenwerking (Dutch)
