# Supplementary material for: Reorganizing the Multidisciplinary Team Meetings in a Tertiary Centre for Gastro-Intestinal Oncology Adds Value to the Internal and Regional Care Pathways. A Mixed Method Evaluation
Source: Int J Integr Care. 2021 Feb 25;21(1):8. doi: 10.5334/ijic.5526 (PMC7908930; doi:10.5334/ijic.5526)
Supplement: Supplementary File 3. — Coding tree reorganization GIO-MDTM. [file ijic-21-1-5526-s3.pdf]

Supplementary file 3. Coding tree evaluation reorganisation Gastro-Intestinal Oncology multidisciplinary team meeting

Major theme	*Minor theme*	*Code*	*Code description*	*Scores*		*Total*
				coder 1	*coder 2*	
Integrated care	Continuity of care	1	The case manager should be a counsellor throughout the entire care pathway, also psychosocial.	14	9	23
	Triage including regional care	2	The GIO-MDTM of the tertiary centre (University Medical Centre) coordinates regional care.	6	8	14
		3	Combining adequate triage of the referral letter with requesting additional information leads to more efficient outpatient visits and GIO-MDTM.	9	4	13
		4	Triage requires expertise of specialist and case manager.	8	5	13
		5	Seeing all patients before GIO-MDTM is not feasible.	3	4	7
		6	Regional secondary centres are sending complex malign and benign cases to expertise in tertiary centre.	3	4	7
		7	The discipline to which the patient is referred (the gate-keeping specialist) is the 'face' of the UMC for the patient.	2	0	2
	Timely treatment plans and treatment	8	The case manager monitors throughput times of the entire care pathway.	14	9	23
		9	The case manager influences diagnostic requests.	15	1	16
		10	GIO-MDTMs lead to customised treatment plan and actions for complex cases.	8	7	15
	Adequate information	11	The case manager accelerates the process to gather information for the GIO-MDTM, often from regional hospitals.	21	5	26
		12	External communication for the tertiary referral works, for instance because of the comprehensive information on the website.	1	3	4
		13	It is not always clear to the patient who the main responsible physician is.	1	1	2
	Patients' wishes	14	Screening on patient wishes with elderly patients (>65 years).	5	10	15
		15	Avoids patients making unnecessary long journeys, also in view of the environment.	1	8	9
		16	Patient satisfaction should be measured.	1	1	2
Added value Multi-disciplinary team meeting (MDTM)	Goals MDTM	17	The policy, goals and value of the GIO-MDTM are important.	7	0	7
	Team performance	18	In the GIO-MDTMs there is always a good team spirit, where colleagues confront each other about undesirable and desirable behaviour.	2	8	10
		19	The physician feels supported by the MDTM.	6	3	9
		20	Unite the case manager and nurse practitioner in the same position.	5	2	7
	Meeting preparation	21	Non-complex cases or 'formalities' and complex cases should be distinguished for efficiency.	8	12	20
		22	Preparation cases mono- or bi-disciplinary for GIO-MDTMs makes them more efficient.	8	3	11
		23	The additional task of specialists to prepare the GIO-MDTM is not scheduled.	4	3	7
	Attendance key members	24	GIO-MDTMs are the moments where all expertise comes together once a week to make an optimal multidisciplinary treatment plan.	21	15	36
		25	Improve structural participation of various specialisms.	3	2	5
	Role of chair	26	The role of the chair of the GIO-MDTM is to monitor the meeting process, summarise discussions and formulate the conclusions.	18	13	31

Major theme	*Minor theme*	*Code*	*Code description*	*Scores coder 1*	*Scores coder 2*	*Total*
		27	The chair of the GIO-MDTM rotates between the surgery and the gastroenterology & hepatology teams.	4	5	9
	Role of gate-keeping specialist in MDTM	28	The gate-keeping specialism is responsible for a good intake and presents the patient in the GIO-MDTM – this is a key role.	15	17	32
		29	For HPB, ‘bilateral’ adjustments are often implemented after the GIO-MDTM without reference in the treatment plan.	2	6	8
	Time to discuss patients	30	Better planning and time management during GIO-MDTM.	9	10	19
		31	There is not enough time for input from case managers in the GIO-MDTM, which hampers discussion on fragile elderly patients and psychosocial aspects.	7	6	13
		32	There is a risk that specialists do not give correct advice due to too much or too little communication - long lists of patients.	2	4	6
	Software database	33	Work more often from EPD during GIO-MDTM.	8	5	13
	Education	34	Knowledge and skills are shared in GIO-MDTM, for instance for training specialists.	3	1	4
Management care pathway	Process and clinical outcomes	35	Lead time on which the physician has no influence.	8	17	25
		36	There is not enough therapeutic capacity, in particular OR capacity for surgery and Endoscopic Retrograde Cholangiopancreatography.	4	0	4
	Slot ‘diagnostics’	37	More awareness is needed of the importance of slots for ‘diagnostic procedures’.	7	7	14
	Slot ‘intake’	38	Better estimation of slots for GIO-intake needed on day of outpatient visit.	5	4	9
	Requirement for MDTM discussion	39	HPB GIO-MDTM can be done ‘on paper’, a GIO-intake is not always needed to make a treatment plan.	3	2	5
	Evaluation of MDTM	40	GIO-MDTM is valuable for quality and an internal second opinion.	6	5	11
		41	There is an increase in the number of patients, which means less time per patient, monitor capacity to prevent quality degradation.	6	2	8
		42	Efficiency: GIO-MDTM stimulates presence.	7	3	10
		43	Reorganisation of the GIO-MDTM did not offer benefits in all care pathways.	5	5	10
		44	Importance of meeting space, more interaction and commitment in U-form.	7	9	16
		45	Case managers are not always invited to the separate meetings on policy and improvement opportunities.	18	6	24
		46	Willingness to improve – a dashboard with indicators gives insight into management results.	5	15	20
	Evidence-based guidelines	47	Discussing all patients from the region in accordance with the guidelines, also the non-complex cases or ‘formalities’.	2	5	7
	Complex	48	Because only complex care is provided in the UMC this means additional workload.	8	4	12
		49	Better indication of patients with comorbidity or fragile elderly patients.	7	3	10
	Clinical trials	50	GIO-MDTMs are important for inclusion of patients in clinical trials.	4	3	7
Total				346	284	630

Legend: coding tree evaluation reorganisation GIO-MDTM:

This coding tree has major and minor themes that were derived from the first research question (effect of reorganisation on integration of care) and second research question (added value as described in perceived benefits and drawbacks) and minor themes derived from researcher's field notes.

If a code was seen as a benefit it is shown in green, 30 codes were identified 418 times (66.3% of the total); drawbacks are shown in red, 20 codes were identified 212 times (33.7%).

The number of scores is given for the first coder, the second coder and the total.

GIO-MDT = Gastro-Intestinal Oncology multidisciplinary team; MDTM = MDT Meeting; HPB = Hepatobiliary; EPD = Electronic Patient Dossier.
