# Supplementary material for: Reorganizing the Multidisciplinary Team Meetings in a Tertiary Centre for Gastro-Intestinal Oncology Adds Value to the Internal and Regional Care Pathways. A Mixed Method Evaluation
Source: Int J Integr Care. 2021 Feb 25;21(1):8. doi: 10.5334/ijic.5526 (PMC7908930; doi:10.5334/ijic.5526)
Supplement: Supplementary File 4. — Patient and tumour characteristics of the care pathways. [file ijic-21-1-5526-s4.pdf]

Supplementary file 4: Tables Patient and tumour characteristics of the care pathway

Table a. Patient and tumour characteristics of the colorectal pathway

	Before reorganisation 2014 (n = 32)		After reorganisation 2015 (n = 34)		Sign.
Age* Mean (sd)	68	(9)	63	(13)	.090
Gender**	n	%	n	%	.088
Female	15	47	23	68	
Tumour localisation***					.804
Colon	6	19	6	18	
Recto-Sigmoid	5	16	5	15	
Rectum	19	59	19	56	
Peritonitis	2	6	2	6	
Abdomen	0	0	2	6	
Tumour size***					.201
T1	2	6	5	15	
T2	1	3	5	15	
T3	11	34	13	38	
T4	5	16	5	15	
Not reported****	13	41	6	18	
Type of diagnosis					
Primary tumour***	12	38	22	65	.114
Locally Adv	2	6	0	0	
Metastases	6	19	5	15	
Recurrence	8	25	6	18	
Restaging	3	0	0	0	
Infection	1	3	1	3	

* = Mann-Whitney-U; ** = Chi^2; *** = Chi^2 Exact; **** Tumour-size not given in MDTM report

Table b. Patient and tumour characteristics of the hepatobiliary pathway

	Before reorganisation 2014 (n = 36)		After reorganisation 2015 (n = 32)		Sign.
Age* Mean (sd)	66	(10)	65	(13)	.863
Gender**	n	%	n	%	.666
Female	15	42	15	47	
Tumour localisation***					1.000
Gall bladder	1	3	1	3	
Pancreas Intra	12	33	11	36	
Ampulla Vateri	1	3	1	3	
Liver	22	61	18	58	
Tumour size***					.549
T1	2	6	3	9	
T2	3	8	4	13	
T3	10	28	7	22	
T4	4	11	7	22	
Not reported****	17	47	11	34	
Type of diagnosis***					
Primary tumour	12	33	18	56	.039
Locally Adv	0	0	1	3	
Metastases	17	47	13	41	
Recurrence	2	6	0	0	
Restaging	3	8	0	0	
Infection	2	6	0	0	

* = Mann-Whitney-U; ** = Chi^2; *** = Chi^2 Exact; **** Tumour-size not given in MDTM report

Table c. Patient and tumour characteristics of the esophagus-stomach pathway

	Before reorganisation 2014 (n = 28)		After reorganisation 2015 (n = 32)		Sign.
Age* Mean (sd)	64	(9)	69	(10)	.050
Gender**	n	%	n	%	.061
Female	9	32	18	56	
Tumour localisation***					.301
Esophagus	24	86	24	75	
Stomach	4	14	8	25	
Tumour size***					.873
T1	4	14	2	6	
T2	4	14	6	19	
T3	14	50	16	50	
T4	5	18	6	19	
Not reported****	1	4	2	6	
Type of diagnosis***					
Primary tumour	26	93	25	78	.802
Metastases	1	4	3	9	
Recurrence	1	4	1	3	
Restaging	0	0	1	3	
Lymphoma	0	0	1	3	
Gist	0	0	1	3	

* = Mann-Whitney-U; ** = Chi^2; *** = Chi^2 Exact; **** Tumour-size not given in MDTM report
